# What helped and hindered implementation of an intervention package to reduce smoking in pregnancy: process evaluation guided by normalization process theory

**DOI:** 10.1186/s12913-019-4122-1

**Published:** 2019-05-09

**Authors:** Susan Jones, Sharon Hamilton, Ruth Bell, Vera Araújo-Soares, Svetlana V. Glinianaia, Eugene M. G. Milne, Martin White, Martyn Willmore, Janet Shucksmith

**Affiliations:** 1 School of Health and Social Care, Teesside University, Borough Road, Middlesbrough, TS1 3BX UK; 2 Institute of Health and Society, Newcastle University, Newcastle upon Tyne, UK; 3 Newcastle City Council, Newcastle upon Tyne, UK; 4 MRC Epidemiology Unit, School of Clinical Medicine, University of Cambridge, Cambridge, UK; 5 Fresh, Smoke Free North East, Durham, UK; 6 PHE North East, Floor 2, Citygate, Gallowgate, Newcastle upon Tyne, UK

**Keywords:** Qualitative research, Normalization process theory, Process evaluation, Complex intervention, Implementation, Acceptability, Smoking, Smoking cessation, Pregnancy

## Abstract

**Background:**

Smoking in pregnancy causes harm to mother and baby. Despite evidence from trials of what helps women quit, implementation in the real world has been hard to achieve. An evidence-based intervention, babyClear©, involving staff training, universal carbon monoxide monitoring, opt-out referral to smoking cessation services, enhanced follow-up protocols and a risk perception tool was introduced across North East England. This paper presents the results of the qualitative analyses, reporting acceptability of the system changes to staff, as well as aids and hindrances to implementation and normalization of this complex intervention.

**Methods:**

Process evaluation was used to complement an effectiveness study. Interviews with maternity and smoking cessation services staff and observations of training were undertaken. Normalization Process Theory (NPT) was used to frame the interview guides and analysis. NPT is an empirically-derived theory, developed by sociologists, that uses four concepts to understand the process of routinising new practices.

**Results:**

Staff interviews took place across eight National Health Service trusts at a time of widespread restructuring in smoking cessation services. Principally interviewees worked in maternity (*n* = 63) and smoking cessation services (*n* = 35). Five main themes, identified inductively, influenced the implementation: 1) initial preparedness of the organisations; 2) staff training; 3) managing partnership working; 4) resources; 5) review and planning for sustainability.

**Conclusions:**

NPT was used to show that the babyClear© package was acceptable to staff in a range of organisations. Illustrated in Themes 1, 2 & 3, staff welcomed ways to approach pregnant women about their smoking, without damaging their professional relationship with them. Predicated on producing individual behaviour change in women, the intervention does this largely through reorganising and standardising healthcare systems that are required to implement best practice guidelines. Changing organisational systems requires belief and commitment from staff, so that they set up and maintain practical adjustments to their practice and are reflective about adapting themselves and the work context as new challenges are encountered. The ongoing challenge is to identify and maintain the elements of the intervention package which are essential for its effectiveness and how to tailor them to local circumstances and resources without compromising its core ingredients.

**Electronic supplementary material:**

The online version of this article (10.1186/s12913-019-4122-1) contains supplementary material, which is available to authorized users.

## Background

Smoking during pregnancy is detrimental to the health of mothers and babies [1–3]. The rate of smoking at delivery in North East England in 2011/12, at study onset, was substantially higher than the UK national average (20.6% vs 13.2%) [4]. Nicotine is highly addictive and smoking behaviours are deeply entrenched, so a combination of cessation measures is required to curb smoking in pregnancy effectively, including psychosocial support, carbon monoxide monitoring and nicotine replacement therapy [5–10]. Previous studies report effective interventions that are educational, motivational, or which offer social support, feedback, use of incentives and counselling for psychological health [6, 11–17]. From these, key elements for cessation approaches aimed at pregnant women have been distilled into National Institute for Health and Care Excellence (NICE) guidance [7, 18–20]. The NICE guidance [8] identifies a number of recommendations, including two key actions for midwives, 1) to identify and 2) to refer for smoking cessation support, pregnant women exposed to tobacco smoke (Table 1).

**Table 1:** Selected recommendations from NICE Public Health Guidance 26 (2010)

NICE (2010) Recommendations	
No.	Description - topic and staff expected to implement
1	Identifying pregnant women who smoke and referring them to *NHS Stop Smoking Services – action for midwives • Assess the woman’s exposure to tobacco smoke through discussion and use of a CO test • Refer all women who smoke, or have stopped smoking within the last 2 weeks, to *NHS Stop Smoking Services
3	Contacting referrals - *NHS Stop Smoking Services
4	Initial and ongoing support - *NHS Stop Smoking Services
6	Meeting the needs of disadvantaged pregnant women who smoke - *NHS Stop Smoking Services
8	Training to deliver interventions - Commissioners of NHS Stop Smoking Services, Maternity services, Professional bodies and organisations, NHS Centre for Smoking Cessation and Training, Other providers of smoking cessation training which meets the national standard.

*Also refers to other publicly funded, free to access, stop smoking services that offer help to quit and operate to the same standard i.e. are evidence-based

Despite the strong evidence base for cessation work with this group and midwives being well placed to intervene with pregnant smokers [21], Beenstock et al. [22] found NICE guidance [8] was not embedded in North East England. Midwives understood that giving cessation advice was integral to their role, but were not convinced it was effective, or that their working practices would allow them to provide effective support [22]. Importantly, they were concerned about damaging their professional relationship with pregnant smokers, mirroring findings in other studies [7, 18–20]. In response, Fresh (the North East regional tobacco control office) [23], with support from the North East Strategic Health Authority (defunct from March 2013), achieved agreement from all eight North East National Health Service (NHS) Foundation Trusts to implement an intervention. Concurrently structures for commissioning and service provision were changing nationally. Responsibility for commissioning stop smoking services moved from the NHS to local government in April 2013, with re-tendering for contracts, resulting in new and different service models being commissioned across the region. In this paper, Stop Smoking in Pregnancy Services (SSPS), will be used as a generic term for the services provided for pregnant smokers to support them to quit, including, publicly funded, free to access, Stop Smoking Services (SSS).

### Intervention

The delivery of a comprehensive, enhanced referral and treatment pathway known as ‘babyClear©’ was commissioned from Improving Performance in Practice (iPiP) [24]. The pathway was based on NICE guidance [8] and developed by iPiP pragmatically (and deliberately) through incorporating knowledge of midwifery experience [25] (Table 2).

**Table 2:** Source of cessation activities in the babyClear© package

Activity	NICE (2010) Recommendation	Pragmatic addition	Beenstock et al. 2012
Universal carbon monoxide monitoring	1		
Provision of CO monitors and lower level of CO threshold for referral (4 ppm)*	1	✓	
Opt-out referral from maternity services	1		
Increase speed of referral by the midwife to the SSPS		✓	
Motivational interviewing by staff who deliver babyClear© package	4, 8		
Target quitting completely, not reduction	1, 8		
Increase speed/strict timeframes within which contact is pursued at each point by the SSPS		✓	
Increase contact with pregnant smokers by the SSPS	1, 3, 4		
Risk perception tool at 12 week dating scan	7, 8	✓	
Offer a variety of accessible SSPS follow up options	3, 4, 7		
**Provide sufficient resources/logistics to deliver the babyClear© pathway			✓
Increased prioritisation of the SS message	8		
Increased buy-in by healthcare staff	8		✓
New discourse between healthcare staff and pregnant women who smoke	8	✓	✓
Increased communication between SSPS and maternity services/integration	1, 4, 6	✓	

*Decision by developers of babyClear© package; NICE guidance 7 ppm

**Recognised as an issue by NICE guidance but not part of a recommendation

**Fig. 1:**
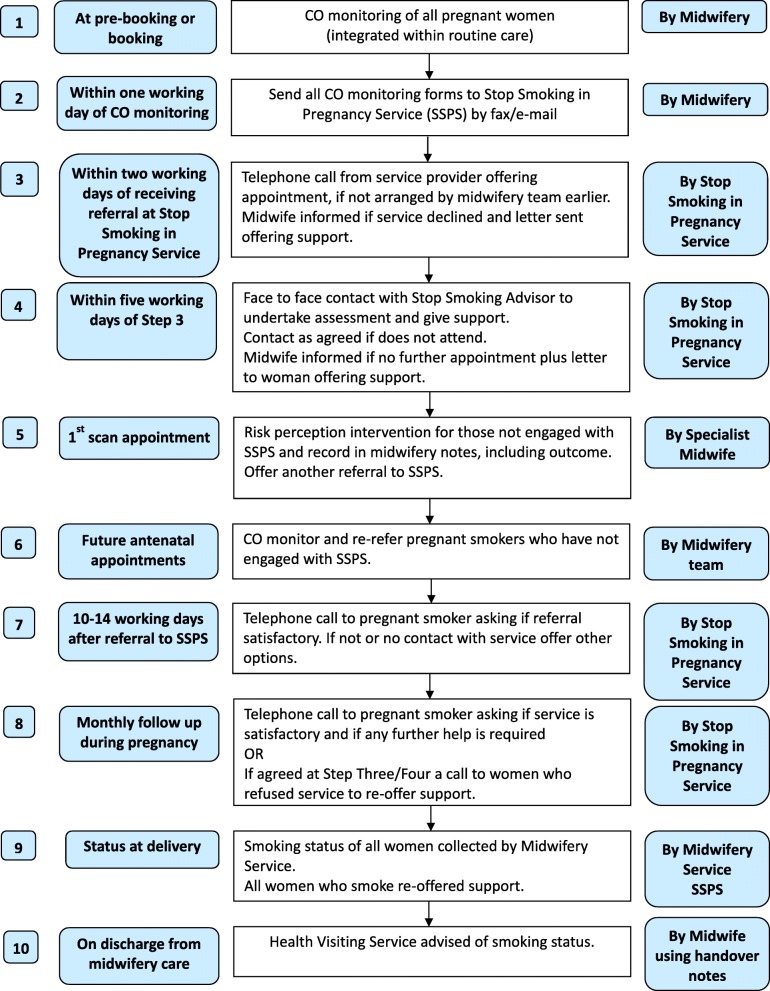
babyClear© referral pathway Reused from Bell et al. in Tobacco Control [5] under the terms of the Creative Commons (CC BY) Attribution Licence. (Amended ‘by’ to ‘my’). Access online at: doi: 10.1136/tobaccocontrol-2016-053476 http://tobaccocontrol.bmj.com/cgi/content/full/tobaccocontrol-2016-053476

The babyClear© intervention (Additional file 1) and referral pathway (Fig. 1) were characterised by: universal systematic screening at booking appointment using, a carbon monoxide (CO) monitor and a threshold of 4 CO parts per million (ppm), opt-out referral to SSPS and an intense follow up regime, utilising a pathway with jointly agreed protocols which strengthened links between midwifery and SSPS.

All maternity and SSPS staff involved in delivering the new pathway of care received training (Additional file 1). The pathway included several additions to NICE guidance (2010) (Table 2), including the risk perception tool (RPT). The RPT was designed to influence those women who were still smoking at the 12-week dating scan: immediately following the scan they received a personalised interview with an experienced midwife, using a computer programme with a visual display linked to a lifelike fetal doll (with umbilical cord, placenta and amniotic sac), to demonstrate the effects of smoking on the fetus. Implementation of the intervention package took place between November 2012 and July 2013, excepting the RPT feature, which was implemented gradually up until data collection ended in January 2015.

### Evaluation

An evaluation of this ‘natural experiment’ was undertaken by a consortium of public health researchers in Fuse [26], who carried out an effectiveness study and cost consequence analysis (reported elsewhere [5]) and a process evaluation. The effectiveness study reported that, after introduction of the intervention (without the RPT feature), referrals to SSPS increased by 2.5 times and the proportion of women quitting smoking by delivery almost doubled; quits during pregnancy were also associated with a clinically important increase in birth weight [5]. Although the time series modelling showed that the new intervention package was effective, it remained unclear how and in what circumstances the measures worked optimally [5]. The process evaluation aimed to develop understanding of how the pathway of care, including the RPT, was implemented and embedded into routine practice. Here we report findings based on the data collected from SSPS and healthcare professionals. The aim of this paper is to present the results of the qualitative analyses, underpinned by NPT, reporting acceptability of the system changes to staff, as well as aids and hindrances to implementation of this complex intervention into routine practice.

### Normalization process theory (NPT)

NPT was chosen because it seeks to illuminate the processes by which staff ‘normalise’ or make routine a new practice [27, 28]. It comprises four main concepts: coherence, cognitive participation, collective action and reflexive monitoring [29] (Table 3). May & Finch see normalization resulting from the operation of these concepts through social interactions [27]. By identifying the concepts at work during implementation, they suggest, it is possible to understand the process.

**Table 3:** Working definitions of Normalization Process Theory concepts [27]

Concept	Key attribute	Working definition
Coherence	Sense-making	The extent to which individuals really understand all the elements of the intervention and the reasons for adopting the new system
Cognitive participation	Engagement	The extent to which individuals believe in or ‘buy into’ the innovation and start to prepare for it
Collective action	Enacting	What happens when the innovation is operationalised
Reflexive monitoring	Appraisal	The act of keeping an innovation under review and adapting it intelligently to changing circumstances

#### Extended NPT

The team that developed NPT has recently identified four factors that dispose guidelines towards being normalised: intervention plasticity, contextual elasticity, coupling and adaptive work which offer further insights into the process [30].

## Methods

### Study design

The process evaluation, uses a qualitative methodology, based on existing MRC guidance [31, 32], that recommended combining quantitative and qualitative methods for evaluating complex interventions, including natural experiments. By using qualitative methods to consider the context and individual perceptions, the guidance suggests, the study’s explanatory power of causality and variation and accuracy of interpretation will be increased, improving knowledge of the intervention package’s transferability and reducing bias [31]. Data were collected between January 2014 – January 2015 and followed implementation as it occurred. The process was recorded by SJ through field notes, observation and semi-structured individual and group interviews. The study itself had several components and included data from pregnant smokers, midwives, trainers, stop smoking service staff, managers and community clinicians. The data from pregnant smokers focused on acceptability of the intervention to women and will be reported elsewhere. NPT informed the interview schedule questions and was used as a framework for data analysis. The Teesside team, JS, SH and SJ, who conducted the qualitative data collection and analysis have backgrounds in public health and nursing. They had no role in the development or implementation of the intervention package; VA-S and MWh gave feedback on the planned pathway of care regarding targeting barriers and facilitators identified by Beenstock et al. [22] and Fresh supported the implementation.

### Participant selection

#### Sampling

Training sessions were observed that took place after ethical approval was gained. Where there was more than one similar session, they were chosen to include different trainers and attendees from a variety of organisations. Interviews took place in services after the intervention package was fully implemented. Data saturation was achieved in some services, but not others, due to delay in implementation; however data saturation was achieved in the dataset overall [33]. Inclusion criteria were: managers of services, trained service delivery personnel, trainers and a representative from Fresh (Table 4).

**Table 4:** Participants - employing organisations and staff roles

Maternity services	Employees of organisations (other than maternity) who provide smoking cessation services	Employees of other organisations
Senior maternity managers in all NHS trusts (*n* = 8)	SSPS senior managers (*n* = 10) (including repeat interviews with participants who moved into new managerial posts (*n* = 2))	Trainers from iPiP (*n* = 3)
Midwives (*n* = 39) (including Public Health midwives (*n* = 2), community midwives (*n* = 20), RPT midwives (*n* = 14), midwifery students (*n* = 3)).	SSPS staff (*n* = 20) (including pregnancy specialists (*n* = 3), advisors (*n* = 10), administrators (*n* = 7)).	Fresh representative (*n* = 1)
Maternity care assistants (*n* = 13)	Public Health nurses (n = 3)	
	Community workers (n = 3)	
	Pharmacy staff (n = 3)	
Total = 60 maternity staff	Total = 39 SSPS staff	Total = 4 other

*SSPS* = Stop Smoking in Pregnancy Services, *RPT* = Risk Perception Tool

All staff who met the inclusion criteria were invited to take part (no incentives were given) and those who agreed, participated (*n* = 103).

#### Recruitment

Senior Trust managers were approached by SJ. Information sheets were cascaded by managers via email to maternity staff, in accordance with ethical requirements. Those who agreed to take part contacted the researcher. Twenty of the 39 midwives interviewed and one smoking cessation specialist/midwife also delivered the RPT. Recruitment procedures were replicated for SSS managers and staff, including generalist and specialist pregnancy advisers, and administrators. Some advisors located in community settings, such as outreach workers, were identified through local government; and other community advisors, such as pharmacy staff, through SJ’s attendance at the training events.

### Data collection

Three qualitative data collection methods were used by SJ, who collected all the data. Firstly, non-participant observation of classroom-based staff training sessions (*n* = 11 sessions). These occurred before implementation of the pathway. Written consent was obtained from the trainer prior to observation of the group and attendees were made aware of the researcher’s presence and purpose. A schedule of observational prompts for each professional role, informed by NPT, was developed to guide data collection (Additional file 2). Knowledge gained from observing the training was used in interviews with staff to explore its relevance and role during implementation. Secondly, a diary was kept by SJ, including field notes, thoughts and reflections, which was completed after fieldwork and during office-based work. Thirdly, interviews, following informed written consent, were conducted in the workplace, except in the case of the trainers, where one was interviewed using a video teleconferencing facility and two in community locations at their convenience. The SSS, commissioned by local authorities, were visited and one response was given via email. Following commissioning decisions, two organisations were re-structured; both were visited before and after. In addition, twenty-three Trust sites were visited, including hospitals and community bases, 4 were visited twice and 2 were visited three times. This includes visits for repeat interviews over time with 2 different organisations and, additionally one telephone conversation. This provided inclusion of views from all staff roles involved in delivering the intervention package, from every NHS Trust and SSPS in the North East region.

Interviews were guided by topic schedules informed by NPT (Additional file 2). One hundred and three participants took part overall. Individual face-to-face interviews, were conducted with 32 participants, including 13 SSS staff, 14 maternity staff, all three trainers, a representative from Fresh and a pharmacist. Sixteen paired interviews also took place with 32 staff. Thirty-seven participants contributed to ten group interviews, which comprised 3–5 participants, allowing for participant preference. Additionally, one participant exchanged emails with the researcher and one participant took part in a telephone interview. Due to delay in introducing the RPT, the longitudinal aspect was modified. Data were collected on completion of implementation or end of data collection period, whichever was sooner. Some participants took part multiple times: three participants were interviewed twice, one participant was interviewed twice and took part in a group interview and one participant was interviewed in a group and answered questions via email. The reasons for repeat participation were: carrying out community and RPT roles, restructuring and changing job roles and looking at change over time. SJ returned to three Trusts, 9–12 months later, to investigate development and sustainability. Two participants had moved to different Trusts and roles at follow up. Individual interviews lasted from 20 to 92 min (average 46), paired interviews from 26 to 82 min (average 44) or larger groups, from 13 to 85 min (average 45). Long interviews tended to be with those who were most engaged with babyClear©, such as local champions, employees of iPip and Fresh. Short interviews were generally in areas where there was less enthusiasm for the implementation. Data were collected in all eight Trusts when organisations were in the early stages of training and implementation. Conversations were digitally recorded unless the participant requested otherwise (*n* = 2), whereupon notes were taken. Data collection continued until every organisation and staff role had participated and no new issues were arising from coding the data [33]. Recordings were transcribed verbatim. Transcripts were returned for member checking on request and anonymised before analysis was undertaken.

### Data analysis

Framework analysis was used to analyse the interview data [34]. This involved five steps: familiarisation by immersion in the data, deciding on a thematic framework (in this case NPT concepts), indexing by coding data to the framework, charting by sorting and summarising the data in each core concept, and finally mapping and interpreting the data in the framework. SJ, JS and SH conducted an initial quality check of the coding by indexing three transcripts independently, followed by ongoing discussion throughout regarding the interpretation of categories as the data emerged. SJ continued to code all transcripts, map and interpret data. JS and SH had ongoing discussions with SJ about the mapping and interpretation work. During this deductive process, it was noted that some additional, cross-cutting themes arose inductively [35]. The relevant data were mapped across then grouped to create the themes presented here (Additional file 3: Table S5). Although NPT informed each of the stages of this study, we have taken a pragmatic decision to present the findings as these practical themes. We have focused on the requirements for normalization in a complex environment, rather than theoretical concepts, to make the findings easily accessible to all. Transcripts from trainers, the representative from Fresh and field notes were used to check the data e.g. for factual accuracy, system details and consistency between researcher observations and staff reports of the training. NVivo 10 software was used to manage data analysis.

## Results

The mapping exercise reported here inductively identified five, cross-cutting themes. The five themes identified major influences on implementation, integration and the extent to which this complex intervention was embedded into routine practice. In both the NHS and local authorities, system change and pressures on budgets in public health services nationally, formed a backdrop to the rollout. During coding it became clear that there was considerable variance between contexts. Staff reported how contexts influenced compliance with NICE guidance (2010) and the intervention protocol. During mapping and interpretation, the ease or otherwise of the operation of the concepts was revealed, which clarified the potential for normalization. For information on how the themes are linked to NPT core concepts see Additional file 3: Table S5.

### Theme 1: preparedness of the organisations

The preparedness of a Trust at organisational level was explored through the NPT concepts of ‘coherence’ and ‘cognitive participation’. Where the culture, ethos and structures of the maternity and SSPS provider organisations were reported as already in line with that of the intervention, i.e. prioritising promotion of smoking cessation, progress towards normalization was easier. For example, in one area where partnership working was well established the SSPS manager commented:

*Midwifery have moved on enormously in the last 5 years in terms of understanding the importance of public health.*

Whereas a SSPS manager from elsewhere said:

*Perhaps the biggest barrier we’ve come across isn’t anything to do with our staff. It’s staff in Maternity Services themselves.*

Similarly, difficulties in implementing the changes as planned were reported where structures were not sympathetic to the intervention’s ethos e.g. in some areas pregnant women were no longer treated by SSS as a group who required specialist attention. All providers of SSPS reported being affected during the study by commissioning changes, service re-structuring and a consequent atmosphere of uncertainty.

Another indicator of preparedness, derived from the data, was belief and attitude towards the implementation; where staff were upbeat, happy with change, open to the introduction of the intervention and believed in its efficacy and benefits, implementation was reported to run more smoothly.

*I think we were really enthused, we really worked with the ethos of wanting to do this … for the women, for the babies, the Public Health agenda and also for us, you know, to be part, to participate in something that has meant health benefits really.* (Senior maternity manager, NHS Trust)

It was noted that the implementation was completed sooner in trusts characterised by maternity staff who recognised their responsibility to prevent stop smoking messages from going ‘off the boil’ (SSPS advisor), took ownership through ‘control of our own team’s quit rate’ (community midwife, team leader) and where there was external support for the intervention (e.g. Commissioning for Quality and Innovation (CQUIN) payments).

*We've got to get down to the nitty gritty of money and because it's one of our CQUIN targets … let's do it properly. So we will always be on the agenda because of the CQUIN targets.* (Community midwife – team leader, NHS Trust)

Clear lines of communication from senior managers, who gave clinical staff ‘permission’ and enabled and trusted them to make the necessary changes associated with the intervention package, were found to be advantageous in achieving successful implementation.

*… there's a Trust drive to get things moving because of the CQUIN target … to me this is more about the underpinning health benefits and that's the way we sell it to the staff, rather than oh, we're just doing it because of the money.* (Matron from early implementing Trust)

Similarly, participants reported normalization as more readily achievable in areas where there had been discussions with representatives of all staff roles, from both services who were going to carry it out, about what it might entail for them. Seeking support from Fresh was thought to be indicative of this attitude.

*We have a good working relationship with Fresh and we understood the rationale for babyClear© and we had done our best to try and adopt what was necessary to do that.* (Manager, SSPS)

In Trusts where champions were appointed who were reported as passionate and motivated about supporting pregnant women to stop smoking, participants recognised how they became opinion-leaders. When they could operate as problem solvers who ensured that training was up to date, offered ongoing support to staff, used feedback effectively to monitor progress, performance manage and improve services, the intervention was reported as more likely to be normalised quickly.

*… (stop smoking lead) kept us really well informed. She comes to our meetings and she is a really good point of contact. If we have got any questions we are on the phone to (her). She always gets back to us and so we are, never feel like we are being out there and on our own, since we got (her). It was very difficult before (she) came, but since (she) came she has really supported and kept us up to date with everything.* (Maternity care assistant, NHS Trust)

### Theme 2: motivational staff training – winning hearts and minds

In line with the NPT concepts of ‘cognitive participation’ and ‘coherence’, it was clear to participants that the training component was essential in helping all staff from both services understand the intervention package and come on board. An increase in referrals following top-up training sessions was confirmed by the effectiveness evaluation [5]. Training, delivered by iPiP, was mandatory for frontline staff; data from observations showed it focused on building their interviewing skills and stressed the importance of consistency of messages to pregnant smokers. It set out clear aims; observations showed that sessions were focused, and were reported as relevant and, in general, as addressing trainees’ concerns. Staff reported that training methods equipped them to deal with real-life scenarios and - critically - offered them a new, positive discourse, including an alternative language, with which to raise stop smoking issues with pregnant women.

*We’ve had this training. There was concern at first. As a team leader, there was not quite opposition … concern … I think that one midwife in particular, said - point blank - she was not doing it. However now we have gone into it, in a tactful way, we have learnt how to deliver it (babyClear©), we have modified our practice around it. I feel it is just part of our every-day (practice) now, isn’t it? Really. It’s not a problem. Actually, it’s a positive.* (Community midwife – team leader, NHS Trust)

*I personally didn’t feel that confident talking about smoking (previously), because I don’t think I understood the whole impact. I think I gained a lot from the training about what the impact of smoking actually is; you know that it’s harmful, but it was how to approach it without alienating the woman, and things like that you know. Because you want the woman to keep coming back for her antenatal care, so it’s important that you form a relationship, and you don’t want anything to sort of spoil that.* (RPT midwife, NHS Trust)

In some cases, staff reported being trained just before implementation, which they felt was ideal; however, when there was a gap between training and using the new knowledge, staff said it was easy to forget it, and lose confidence. It was observed to be a significant challenge to the training programme to make the content adaptable to local contexts (e.g. areas varied widely in size, geography, population demographic and service delivery model) and made taking ‘collective action’ more challenging.

### Theme 3: managing partnership working

Participants expressed how partnership working of a high standard between maternity services and SSPS was required to take ‘collective action’; however, there were different approaches across the region (Additional file 4: Table S6). Partnership working (e.g. frequent face-to-face meetings between members of both maternity services and SSPS) encouraged a growing familiarity between them and an ethos that fostered the development of efficient feedback loops. Participants explained how feedback was an important form of ‘reflexive monitoring’.

*The whole success of babyClear© is the kind of linking with maternity services, because from the stop smoking side of it, it adds credibility to the message if it’s coming from a midwife, and, you know … it incorporates smoking (cessation) as standard practice.* (Pregnancy specialist advisor, SSPS)

Central to ‘cognitive participation’ is sharing the tasks along the pathway. Working in partnership, it was reported, requires a clear channel of communication both within and between services. Organisational structures that embedded these channels were reported to promote normalization of the intervention. Where clear communication was present, study participants reported that missing data were reduced, opt out rates were minimised, high rates of consent to contact women were provided, quick returns of referral forms and high standards of form completion were encouraged, deadlines for follow up criteria were met, women were re-referred where appropriate and any changes (e.g. staff turnover or service alterations) were communicated in a timely manner. The converse was reported to hinder progress:

*We need the referrals; we can only be as good as the referral that comes in, and there’s quite a few coming in which aren’t very good quality. So, we’re having to second guess whether this person smokes, doesn’t smoke, wants to stop smoking, or doesn’t want to stop smoking.* (Administrator, SSPS)

Once partnership working had allowed for ‘cognitive participation’ and ‘collective action’, informal appraisal, an element of ‘reflexive monitoring’, was able to reveal that stop smoking follow up provision needed to include a) the capacity to absorb the increase in referrals; b) an administrative structure to accept and process referrals, offer first contact to engage women successfully, book appointments, answer queries satisfactorily, offer support where appropriate, consistently re-contact women as per pathway; and c) offer a variety of options (choice of settings and providers, convenient, flexible) and pass women on to advisors knowledgeable about smoking in pregnancy. Some service structures were reported to act as barriers to these processes (e.g. sending SSPS calls which hid the caller’s number).

To monitor progress and enable ‘reflexive monitoring’, participants said that a data management system, operating to a high standard, was required. Participants explained that these information systems were required to capture data on the implementation and delivery of the intervention, enter data at source, create efficient and effective intra and inter agency feedback loops, allow for performance management, promote continuity of care and enable payments to providers. QuitManager© (a data management software package) [36] was recommended by iPiP.

*QuitManager© does allow you to have that robust data; we can examine it on an individual level, across the team level and break it down by attendance, break it down by opt-out, break it down even by setting quit dates, you know. Is there less of a disengagement further down the line? But without that data it's very difficult to do that and it's difficult to tailor the support for maternity staff.* (Pregnancy specialist advisor, SSPS)

‘Reflexive monitoring’ showed that audit and feedback were not always systematised and thus subject to local variability and ad hoc development.

### Theme 4: resources available to support the intervention

Resources affected the ‘collective action’ needed to operationalise the intervention. Some resources were provided by Fresh [5] (Additional file 1) but there was also significant organisational variance in provision (Additional file 5: Table S7

*It’s the commissioner’s decision. They kept saying that we were going to get QuitManager© and we were meant to get it in April two years ago. Then we were meant to get it in April last year. And then we were told we’re going to get it in October this year.* (Administrator, SSPS)

The data showed that where resource conditions could not be met, participants reported that it was harder to implement and sustain the intervention.

### Theme 5: review and planning for sustainability

This theme concerns the NPT concept ‘reflexive monitoring’. During the initial embedding phase, participants stated there was a need to make opportunities for regular, frequent review of the implementation process. For example, the stop smoking lead in SSS-led Trusts met monthly with community maternity teams, with midwives delivering the RPT, with SSPS administrative staff and with whoever was delivering stop smoking follow up. The aim was to discuss progress, problem solve and celebrate success.

Participants talked about performance management of maternity staff, usually by the stop smoking lead, being required to monitor progress and promote embedding, while adhering to the intervention standard. This included assessment of community maternity teams and individual midwives regarding CO monitoring, number of opt-outs, engagement with the SSPS of pregnant women who smoke, as well as the number of quits. Regular dissemination of these results to maternity service staff, and results relevant to SSS staff, through robust communication channels, was reported as highly desirable.

*Part of my role is responsibility for the monthly feedback to the midwives, so what I do is I produce ... a monthly report on the CO referrals that we’ve received, the numbers. I send that back to (senior manager A) and (senior manager B), and I also do one on the referral outcomes. Now what I’ve suggested is that on a monthly basis I do more of an individualised one for the team leaders, so that it actually gets rather than one report going to (senior manager) to then be disseminated to team leads.* (Specialist pregnancy advisor, SSPS)

Once it was established that the intervention had been normalised, i.e. different ways of working had become routine, participants stated that a less frequent review cycle was required. The stop smoking lead role was reported to change to one of maintenance; however, responsibility remained to ensure that training was regularly updated, performance remained high, the stop smoking agenda was kept to the fore and the intervention was successfully sustained to the observed standard in the training.

## Discussion

### Summary of key findings

**Fig. 2:**
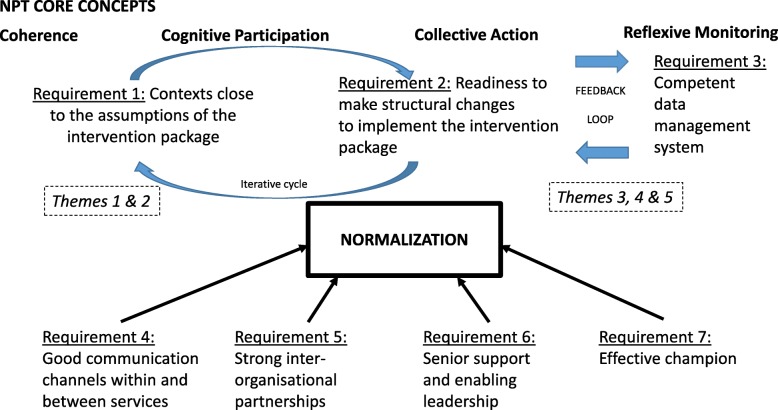
Summary of key findings and their relationship to NPT

Five key themes were identified in aiding or hindering normalization: 1) preparedness of the organisations, 2) staff training, 3) managing partnership working, 4) availability of resources and 5) review/ planning for sustainability (Fig. 2).

Normalization was more likely where these five themes supported ‘coherence’ and ‘cognitive participation’, leading to requirement 1: contexts close to the assumptions of the intervention presented in training, and/or the delivery of the stop smoking message already prioritised and well-integrated (Themes 1 & 2).

‘Collective action’, identified requirement 2: readiness to make structural changes to enable the implementation and ensure continued and increasing ‘coherence’ and ‘cognitive participation’ (Themes 3, 4 & 5). These included requirement 3: a competent data management system (which was introduced in some areas only, in partnership with SSPS), requirement 4: good communication channels within and between services and requirement 5: strong inter-organisational partnerships (Theme 3).

Making structural changes required support from the organisations and staff. Part of the challenge and the complexity lay in the different service delivery models (Theme 3) and organisational contexts (Themes 1–5) and how they had (or had not) supported these structural changes. Different contexts were associated with differing levels of normalization of this standardised protocol. Making structural changes, at this time, was complicated by the concurrent changes to commissioning responsibilities and subsequent provision decisions. Senior support and enabling leadership (Requirement 6) across all NPT core concepts was required to make progress (Theme 3).

The stop smoking lead or champion, requirement 7, was a key role in preparing maternity organisations (Theme 1), motivating and training staff (Theme 2) and managing partnership working (Theme 3). They were able to link services, people and logistics, bring people on board and drive the normalization process forward (Theme 3). Organisational variability and instability challenged implementation and acceptability to staff; however, an effective stop smoking lead maximised the benefits and minimised the challenges. Furthermore, it might be assumed that maternity and SSPS services and staff are similar within the NHS, however they are variable, and judgements must be made as to how similar another local context is to the study reported here and therefore the transferability of findings.

### Relationship to existing knowledge

#### What aids and hinders implementation of guidelines

This study’s findings reflect what is already known, that external and internal organisational contextual factors are important, and so is how individuals think and work, as these have implications for the implementation as well, and cannot be divorced from the progress of the normalization process [30]. A variety of empirical studies using NPT has already illustrated how individual staff affect the likelihood of an innovation becoming routinely and successfully embedded in practice [37–40]. Studies using NPT to explore the adoption of evidence-based guidelines in other clinical areas, especially when implementation is problematic, have also been conducted [41–45]. Morriss [44] found that recommendations have to be workable and properly resourced, or they are less likely to happen. A person to champion the changes and drive through uptake of the recommendations has been found to be helpful [44, 46]. Time to train, as well as trainers using effective techniques, and the use of a selection of approaches is considered most likely to change staff behaviour [44]. These results accord with those found in this study.

Bamford et al. [38] highlighted the centrality of the concept of coherence – staff fully comprehending and prepared to invest in implementing the guidelines – plus the need to equip staff with knowledge, offer institutional support and establish a review process, so that benefits can be evidenced. Similarly Bouamrane & Mair [42] found that coherence was important from the outset, the guidelines needed to be workable and implementing them required appropriate and adequate resources. Sustained engagement, allied with a workable solution, was more likely to succeed if frontline staff were involved at the design stage [42], as they were in the babyClear© work, through consultation for the Beenstock et al. study [22] and use of this data by Fresh to develop the intervention specification.

In relation to this study, plasticity and contextual elasticity were exemplified in the preparedness of various service providers and the receptiveness of their structures to adapt the intervention to the local context [30]. However, where intervention components with low plasticity (‘non-negotiables’) were involved, such as insisting on a midwife to carry out the RPT, opportunities for normative and relational restructuring were limited. May et al. [30] call this interdependence between actors, intervention components and contexts, ‘coupling’ and would suggest that the degree of coupling was indicative of the relative outcomes, as it reflected how easily staff were able to restructure and normalise the intervention.

May et al. [30] have taken a timely step forward, borne out of the studies that have employed NPT to date. In particular, their paper explores the contested issues around what is essential and what is desirable within a complex, practice-based intervention, and how multiple factors operate during the implementation process to allow flexibility but maintain fidelity through coupling [30]. This was a fundamental issue around the implementation of the babyClear© package. The effect of contextual factors on all levels of implementation has often been neglected in reports of experimental interventions carried out under controlled conditions [47]. It is of critical importance that explanatory work is conducted to understand the causal processes when operating in complex, adaptive systems.

### Strengths and limitations

#### Strengths

This is the first study to explore how an intervention, babyClear©, that has proven effective at decreasing smoking in pregnancy, was implemented in practice; and the first to use NPT to do this. A range of methods of data collection, enabling triangulation of data sources and analyses, were used. Large samples of participants from all NHS trusts in the region and all staff types and grades who were involved in designing, commissioning and delivering the intervention participated. Therefore all relevant professional perspectives are included in the data. Thematic saturation for each set of interviews was achieved and a sample of training sessions observed. Research was underpinned using NPT, ensuring theoretical rigour in both data collection and analyses. The outcomes of the effectiveness study were not known at the time of analysis, and did not influence the process evaluation findings. Inductive analysis was used to reveal applied themes not directly related to NPT constructs.

#### Limitations

The study was mostly cross-sectional, rather than longitudinal, although interviews were repeated with a few staff in early implementing Trusts. The intervention is available nationally, but the context of implementation in the North East might mean that the findings should be applied with caution elsewhere. The effectiveness of the complete intervention, including the RPT, remains untested, due to delays in implementation of the RPT component across all participating Trusts. However, service provider responses were assessed around all elements of the intervention, including the RPT, in those sites where it was implemented. Service delivery models varied much more widely in both maternity and SSPS than had been anticipated. Maintaining intervention fidelity therefore became a major challenge for the services.

### Implications for policy and practice

To sustain the core intervention - CO monitoring, opt-out referral and follow-up - there is a requirement to identify and understand, in what circumstances elements of the babyClear© package operate, and which can be tailored to local circumstances and resources, without destroying the integrity of the original model. Local contexts were very different and there is evidence that some flexibility and tailoring is helpful in a ‘real world’ setting. Understanding of the process of normalization of the babyClear© package can assist practitioners in implementing best practice guidelines more effectively and at scale.

## Conclusions

NPT was used to show that babyClear© offers an evidence-informed, intervention package that is acceptable to staff and which can be implemented effectively. It is predicated on producing individual behaviour change in pregnant women who smoke, but does this, in large part through reorganising and standardising maternity and SSPS systems and upskilling staff. Re-structuring those systems requires staff to understand the essence of the new intervention package (coherence), to believe in it and find it congruent with their other work and professional standards (cognitive participation), to set up and maintain practical adjustments to their work practices (collective action) and be reflective about adapting themselves and the work context as new challenges are encountered (reflexive monitoring). Fundamental requirements included active senior management facilitation of change, close partnership working and clear inter and intra agency communication channels, the appointment of an effective champion, high quality training and access to sufficient resources to manage data and implement the measures.

## Additional files

Additional file 1: **Appendix 1.** Intervention description. (DOCX 145 kb)

Additional file 2: Topic guides schedules used for observations, individual and group interview data collection. (DOCX 21 kb)

Additional file 3: **Table S5.** Key themes and their relation to Normalization Process Theory concepts. (DOCX 23 kb)

Additional file 4: **Table S6.** Leadership and management responsible for implementation of the intervention. (DOCX 23 kb)

Additional file 5: **Table S7.** Key resources and organisational variance. (DOCX 22 kb)

## Abbreviations

CO: Carbon monoxide
CQUIN: Commissioning for Quality and Innovation
NHS: National Health Service
NIHR: National Institute for Health Research
NPT: Normalization Process Theory
RPT: Risk Perception Tool
SPHR: NIHR School for Public Health Research
SSPS: Stop Smoking in Pregnancy Services, generic term for the services provided for pregnant smokers to support them to quit, including – but not exclusively - publicly funded, free to access, Stop Smoking Services that offer help to quit and operate to the same standard i.e. are evidence-based
SSS: Local, publicly funded, free to access, Stop Smoking Services
