# Supplementary material for: What helped and hindered implementation of an intervention package to reduce smoking in pregnancy: process evaluation guided by normalization process theory
Source: BMC Health Serv Res. 2019 May 9;19:297. doi: 10.1186/s12913-019-4122-1 (PMC6509824; doi:10.1186/s12913-019-4122-1)
Supplement: Supplementary file 1 — Appendix 1. Intervention description. (DOCX 145 kb) [file 12913_2019_4122_MOESM1_ESM.docx]

**Appendix 1: Intervention description**

Reused from Bell et al. in Tobacco Control [5] under the terms of the Creative Commons (CC BY) Attribution Licence. Access online at: doi: 10.1136/tobaccocontrol-2016-053476 <http://tobaccocontrol.bmj.com/cgi/content/full/tobaccocontrol-2016-053476>

This description is based on information extracted from the service specification used for the tendering process for the intervention, supplemented by materials provided by the Tobacco Control Collaborating Centre, contemporaneous records of training dates and numbers, and observations of training sessions by the research team. It follows as far as possible the elements of the TIDieR checklist (Hoffman et al 2014).

1. **Name:** babyClear^©^
2. **Rationale**

**Extract from service specification developed by Fresh North East for the tender process (March 2012)**

The dangers of smoking during pregnancy are well-established. Evidence shows that it is associated with an increased risk of miscarriage, stillbirth and low birth weight, resulting in an estimated 360 foetal deaths each year in the North East alone. The North East currently has the highest smoking rate at time of delivery (20.6%) of any region in England (the national average is 13.2% and in London, the rate is 6.0%).

Reducing the number of women smoking during pregnancy is one of the key smoking outcomes contained within the Public Health Outcomes Framework for England 2013-16, with an indicator on smoking status at delivery being set. “Healthy Lives, Healthy People: A Tobacco Control Plan for England”, published in March 2011, also sets out the Government`s ambition to reduce the rates of smoking in pregnancy to 11% or less by 2015.

Current NICE public health guidance No. 26 on *“Quitting smoking in pregnancy and following childbirth”* identifies a number of key actions for midwives to take in helping to support pregnant smokers to quit, including routine carbon monoxide (CO) monitoring at booking and referral of all identified smokers to Stop Smoking Services. NICE estimates the cost of smoking during pregnancy for an average primary care trust (PCT) to be up to £575,000 per annum.^1^

NHS Stop Smoking Services provide free, professional and cost-effective support to pregnant women who are interested in quitting. However, North East Stop Smoking Services currently do not reach the majority of pregnant smokers. In 2010/11, 6,348 pregnant women were recorded as smoking at delivery across the North East. Over the same period only 1,792 pregnant women set a quit date with an NHS Stop Smoking Service (SSS). There is also significant variation across the region in terms of referral pathways and in the number of pregnant women accessing and quitting smoking with NHS SSS support. It is imperative that we significantly increase the number of women being referred into services, whilst increasing the capacity of those services to support more pregnant women to stop smoking. We must ensure that all women who smoke during pregnancy are identified, referred, and given the best, most effective support to quit.

Following extensive research and development work with North East midwives in 2011^2^ it was established that some of the key barriers faced by these professionals when discussing smoking include:

- An unfounded belief that delivering stop smoking interventions and referral into services will not have an impact on individual behaviour
- Lack of access to carbon monoxide monitors, and a lack of confidence in using them correctly
- Shortage of time to discuss smoking issues in detail
- Absence of a consistent script or key messages to deliver to clients
- Concerns over the potential to damage relationships with pregnant woman.

Subsequent evaluation of current referral pathways has also highlighted that even where pregnant smokers are being referred into NHS Stop Smoking Services (SSS), only a minority of these women go on to set quit dates. Of those that do make a formal quit attempt with NHS support, only 37% go on to be successful quitters after 4 weeks (2011/12 NHS SSS data). It is therefore imperative that the North East makes every attempt to ensure that:

- All NE pregnant smokers are identified at time of first booking appointment with their midwife through the systematic biochemical testing of carbon monoxide (CO) levels
- All pregnant women recording over 4ppm of CO are referred into NHS Stop Smoking Services at booking for further assessment
- Systems are in place for midwives to continue to raise smoking as an issue at subsequent appointments and refer into NHS SSS as required
- NHS SSS administrative teams employ systematised and evidence-based approaches to convert these referrals into appointments kept and that the service implements an effective lead management system
- The capacity and quality of NHS SSS is sufficient to convert as many pregnant smokers into non-smokers as possible. The service also needs to routinely provide support to partners/key family members of pregnant smokers as appropriate.

1. Quitting Smoking in Pregnancy and Following Childbirth – Costing Tool NICE Public Health Guidance 26: 2010

2. Beenstock J. et al. Impementation Science; 2014:

1. **Elements of intervention**

**Table 1.1 Planned and delivered components of the intervention**

| **Element** | **Who, how, where** | **How much** | **Tailoring** | **Modifications during delivery** | **How well delivered** |
| --- | --- | --- | --- | --- | --- |
| Materials and resources:   - Calibrated carbon monoxide monitors, mouthpieces and D pieces for all NE community midwives - Public-facing leaflets on the dangers of smoking in pregnancy - Referral pads - “Tommys” guide on smoking and pregnancy - Bedfont foetal carbon monoxide monitors and software, and accompanying foetal dolls for risk perception intervention | Provided to all eight Trusts by Fresh (North East tobacco control office).  babyClear© branded resources (CO monitors, referral pads) supplied by TCCC | Sufficient resources for all community midwives employed by the eight Trusts for one year: 330 CO monitors plus consumables, 410 Tommy’s leaflets  15,000 of each of three public facing leaflets  2400 referral pads  20 foetal CO monitors and dolls | The number of CO monitors was tailored to number of deliveries and staff employed in each Trust.  Leaflets and pads – same amount sent to each Trust  Public facing leaflets were modified to include local Stop Smoking Service (SSS) contact numbers | Contact numbers were changed where SSS delivery changed | Provided as planned, but equipment for risk perception intervention not used during the timeframe of this evaluation |
| Training: two hour midwife training sessions (see figure 2) | TCCC to deliver two-hour sessions on NHS sites across the North East to enable midwives (particularly community based midwives) to deliver brief (3-minute) interventions to pregnant smokers at time of first booking appointment. These will focus on systematically implementing NICE guidance on routinely biochemically establishing smoking status by means of a carbon monoxide (CO) test, and automatically referring into NHS Stop Smoking Services where maternal smoking, or exposure to CO, is indicated. Sessions will also cover resources to be used, referral pathways into NHS SSS, and follow-up at subsequent appointments | A minimum of 36 sessions across the region | Number of sessions in each locality tailored to number of midwives. | Additional training sessions were needed to cover midwives who could not attend initially scheduled training, and to cover new staff members. Some sessions were delivered by local staff (trained SSS mentors) using the TCCC materials | Training content was largely delivered with fidelity, but not to the original timetable for roll out. A total of 40 training sessions were delivered including 413 midwives. |
| Training: one day risk perception training sessions | TCCC to deliver one day sessions on NHS sites across the North East to speciality midwives to deliver a more intensive, health-focussed “Risk Perception” intervention at the time of the first dating scan (approx. week 12 of pregnancy) to women who continue to smoke throughout pregnancy. This will enable a cohort of midwives to deliver this intervention to women who have previously declined support or continue to smoke, and will highlight the potential harm being caused to the foetus through continued smoking. The sessions will also reiterate the processes for referring pregnant smokers into NHS Stop Smoking Services. | A minimum of four | None | None | Training content and amount delivered as planned, but severely delayed in many Trusts. Implementation was too late to affect quit rates among the pregnancies included in the evaluation  44 midwives eventually trained |
| Training: existing Stop Smoking Advisors | TCCC to deliver one day skills refresher sessions for existing Stop Smoking Advisors working with pregnant women in order to ensure that they are following the latest evidence-based information in delivering on-going behavioural stop smoking support. | A minimum of four | None | None | Content delivered as planned. Four sessions plus one mop up session delivered to around 95 advisors. |
| Training: new Stop Smoking Advisors | TCCC to deliver two day skills development sessions for Stop Smoking Advisors not currently working with pregnant women in order to ensure that they are following the latest evidence-based information in delivering on-going behavioural stop smoking support. | A minimum of four | None | None | Four sessions delivered covering 54 stop smoking advisors working in a variety of roles. Additional sessions (n=3) designed for pharmacists giving smoking cessation advice were delivered in two areas. |
| Training: Stop Smoking Service administrative support staff | TCCC to deliver one day skills sessions for SSS administrative support staff (or any staff involved in lead management locally) on call-handling and customer relationship management techniques and systems. These will specifically focus on improving engagement with pregnant smokers who have been referred into NHS SSS and converting a higher proportion of these leads into appointments made and kept. | A minimum of four | None | One session only delivered, as it was agreed it was more efficient to bring all relevant staff together | Content delivered as planned to 31 staff. |

**4 Behaviour change techniques identified in the evaluated intervention**

**Table 1.2. Behaviour change techniques and expected mechanism of action**

| **Procedure/materials and resources** | **Behaviour Change Technique (s)**  Adapted from BCT V1 and the Taxonomy for individual behavioural support for smoking cessation (Michie et al 2013 and Michie, Hyder, Walia & West, 2011) | **Expected Mechanism of Action** (based on the Theoretical Domains Framework (TDF), Cane et al, 2012) |
| --- | --- | --- |
| **Overall description of training session for midwives** | | |
| Training: two hour midwife training sessions (see figure 2)  Training on:  routinely biochemically establishing smoking status by means of a carbon monoxide (CO) test, and automatically referring into NHS Stop Smoking Services where maternal smoking, or exposure to CO, is indicated. Sessions will also cover resources to be used, referral pathways into NHS SSS, and follow-up at subsequent appointments. | Instruction on how to perform the behaviour  Modelling  Action Planning  Restructuring the physical environment | Knowledge  Skills  Motivation/intentions  Goals  Environmental context and resources  Social/professional role and identity |
| ***Materials/resources provided/used during training for midwives*** | | |
| Calibrated carbon monoxide monitors, mouthpieces and D pieces for all NE community midwives | Restructuring the physical environment (by providing CO monitors plus consumables for all midwives) | Environmental context and resources  Memory attention and decision processes |
| Referral pads. As part of roll-out there was a standard template for a referral pad to ensure the same level of patient information across the 8 different trusts. This referral pad has an "opt-out" signature box. Each trust adapted it accordingly, leaving the core details on. The details captured in the referral pad aimed at facilitating the sharing of information between Maternity teams and SSS teams. | Action planning (for contact with pregnant woman: when and how)  Monitoring of others behaviour  Restructuring the physical environment (by providing referral pads) | Goals  Environmental context and resources  Memory attention and decision processes  Behavioural Regulation |
| Tommy`s leaflet. This leaflet had key information for maternity staff on how to introduce the issue of smoking with pregnant woman. The leaflet contained some key facts on smoking in pregnancy. This was handed out to all staff who attended basic training. | Provide information on consequences of smoking behaviour  Boost Motivation  Provide information on consequences of smoking cessation  Facilitate barrier identification and problem solving  Explain the purpose of CO monitoring  Build general rapport  Tailor interactions appropriately | Knowledge  Skills  Motivation/Intentions  Behavioural regulation  Communication skills  Social/professional role and identity  Beliefs about capabilities  Beliefs about consequences  Memory attention and decision processes  Social influences |

| **Overall description of the intervention provided to pregnant women** | | |
| --- | --- | --- |
| ***Materials/resources provided to pregnant women*** | | |
| Leaflet on harms of smoking: ‘When you smoke, your baby smokes: all the important facts you need to know about the dangers of smoking in pregnancy’. To be given out to all pregnant smokers as part of booking/1st appointment. Contains information and details of local SSS. This leaflet was personalised 6 times (one per each SSS) to add the local SSS number and the local branding. All other information was identical (developed by Gingernut creative with BabyClear using focus groups of pregnant smokers). | Credible source (NHS Foundation Trust)  Provide information on consequences of smoking (smoking and passive smoking effects on pregnancy, unborn babies and babies and children)  Provide instructions on how to perform the behaviour (contact of the Stop Smoking Service)  (provide instructions on) Restructuring the social environment  Facilitate barrier identification and problem solving  Boost motivation  Fear appeal (Image of a very poorly baby; image of a foetus surrounded by smoke; front image where an opening comes from the tummy and smoke comes out) | Knowledge  Skills  Motivation/intentions  Goals  Behavioural regulation  Beliefs about capabilities  Beliefs about consequences  Memory attention and decision processes  Social influences  Emotion |
| Leaflet on the use of Nicotine Replacement Therapy: ‘Now is the Right Time (NRT): you can use NRT in pregnancy and it is free’. To be handed out to smokers at 1st appointment. This leaflet was personalised 6 times (one per each SSS) to add the local SSS number and the local branding. All other information was identical (developed by Gingernut creative with BabyClear using focus groups of pregnant smokers). | Credible source (NHS Foundation Trust)  Advise on stop-smoking medication (NRT)  Provide information on consequences of smoking during pregnancy | Knowledge  Motivation/intentions  Goals  Beliefs about consequences  Memory attention and decision processes  Behavioural regulation |
| Leaflet-‘ Smokefree Families: Protect your family. Keep your care and home smokefree.’ This leaflet targets the importance of smokefree homes/impact of secondhand smoke. This is not localised, but just generic. To be handed out to all pregnant smokers or women living in households where others smoke. | Credible source (Fresh).  Provide information on consequences of smoking (of second-hand smoke).  Provide information on consequences of being smoke free  Instructions on how to perform the behaviour (how to make a smoke free home)  Facilitate barrier identification and problem solving  (Instructions on how to) restructure the social environment.  (Instructions on how to) restructure the physical environment.  Identification of self as a role model. | Knowledge  Skills  Motivation/intentions  Goals  Environmental context and resources  Beliefs about consequences  Social influences  Behaviour regulation  Memory attention and decision processes  Social/professional role and identity |

**References**

Hoffmann, T.C., et al., *Better reporting of interventions: template for intervention description and replication (TIDieR) checklist and guide.* BMJ, 2014. **348**.

Michie, S., et al., *Development of a taxonomy of behaviour change techniques used in individual behavioural support for smoking cessation.* Addictive Behaviors, 2011. **36**(4): p. 315-319.

Michie, S., et al., *The Behavior Change Technique Taxonomy (v1) of 93 Hierarchically Clustered Techniques: Building an International Consensus for the Reporting of Behavior Change Interventions.* Annals of Behavioral Medicine, 2013. **46**(1): p. 81-95.

Cane, J., D. O’Connor, and S. Michie, *Validation of the theoretical domains framework for use in behaviour change and implementation research.* Implementation Science, 2012. **7**(1): p. 1-17.

**Figure 2: Example of training session programme**
