# Supplementary material for: What helped and hindered implementation of an intervention package to reduce smoking in pregnancy: process evaluation guided by normalization process theory
Source: BMC Health Serv Res. 2019 May 9;19:297. doi: 10.1186/s12913-019-4122-1 (PMC6509824; doi:10.1186/s12913-019-4122-1)
Supplement: Supplementary file 2 — Topic guides schedules used for observations, individual and group interview data collection. (DOCX 21 kb) [file 12913_2019_4122_MOESM2_ESM.docx]

# TOPIC GUIDES

# for observations, individual and group interviews

**PROMPTS for observation of training sessions**

**(2-hour basic training – midwives / midwifery assistants)**

COHERENCE

Does the training help the attendees to gain an understanding of the reasons behind the intervention, including its various elements?

Does it explain the relevance to their practice?

COGNITIVE PARTICIPATION

Do the attendees engage with the training?

How do the trainers engage the attendees?

Are the attendees motivated to implement the intervention?

Are there barriers to cognitive participation? E.g. Do the attendees have reservations about the intervention? How do the trainers handle this?

COLLECTIVE ACTION

Is there a sense of collaboration / team working / across boundaries (e.g. across roles, geographical bases) in the session?

Are questions raised about how this will work out in practice? How other staff may be affected? Practical considerations?

REFLEXIVE MONITORING

Are any plans for future reviewing of the intervention discussed?

What are people’s reactions to considerations about the long-term sustainability of the changes initiated by the intervention?

Are any reservations expressed?

GENERAL

How closely the training session meets its stated objectives, whether the content is delivered as planned, and the responsiveness of the participants to the training session and material.

**PROMPTS for observation of training sessions**

**(risk perception tool training – midwives)**

COHERENCE

Does the training help the attendees to gain an understanding of the reasons behind the ‘tough love’ intervention?

Does it explain the relevance to their practice?

COGNITIVE PARTICIPATION

Do the attendees engage with the training?

How do the trainers engage the attendees?

Are the attendees motivated to implement the intervention?

Are there barriers to cognitive participation? E.g. Do the attendees have reservations about delivering the ‘tough love’ intervention? How do the trainers handle this?

COLLECTIVE ACTION

Is there a sense of collaboration / team working / across boundaries (e.g. across roles, geographical bases) in the session?

Are questions raised about how this will work out in practice? How other staff may be affected? Practical considerations?

REFLEXIVE MONITORING

Are any plans for future reviewing of the intervention discussed?

What are people’s reactions to considerations about the long-term sustainability of the changes initiated by the intervention?

Are any reservations expressed?

GENERAL

How closely the training session meets its stated objectives, whether the content is delivered as planned, and the responsiveness of the participants to the training session and material.

**TOPIC GUIDE for individual/group interviews – midwives and SSPS staff**

COHERENCE

What were your thoughts about the intervention before you came to the training?

Prompt: How clear was your sense of the purpose of the intervention beforehand?

How clear was the intervention made to you in the training?

Prompt: When you finished the training was what you had to do clearly defined in your mind?

Do you have any particular comments you would like to make about the training?

COGNITIVE PARTICIPATION

Has everyone joined in when you have taken it back into practice?

Prompt: Do you think everyone has taken it on board as an idea? Do people think it is a good idea?

COLLECTIVE ACTION

How practical has it proved to be in the work environment?

Prompt: Are there times when it is difficult to implement it?

Did it work out the way you expected?

Prompt: How acceptable was the design? Was it possible to keep to the design when you came to deliver the intervention?

For midwives only: Explore the procedures for tackling referrals who did not attend SSPS when they return for their next appointment; ask RPT midwives about their experience of delivering the ‘tough love’ message to women who do not take up referral to SSPS.

Using examples given by the individual/group ask if any changes have been introduced to address implementation issues / ways in which they have had to adapt their practice.

REFLEXIVE MONITORING

Are you reviewing the changes in any way?

Prompt: How do you know if the intervention is making any difference?

How do you see it working out in the future?

For midwives only: Prompt: Is it likely to continue as it is? Are people going to struggle to continue to give a brief intervention or come in with the ‘tough love’ approach for any reason?

Are there any elements of the intervention that you foresee will be readily sustainable?

Are there any elements of the intervention that you foresee will be difficult to sustain?

Are there any developments / changes that you would like to see into the future?

Prompt: Anything that you feel doesn’t work well at the moment – how might it be improved?

How do you see the smoking cessation agenda evolving (locally, nationally)?

Prompt: What impact is that likely to have on your practice?

**TOPIC GUIDE for interview – maternity service managers**

COHERENCE

What is the purpose of the intervention?

Do you see any problems with increasing smoking cessation rates among pregnant women?

Did you expect this intervention to address them? If yes, to what extent is it doing so?

COGNITIVE PARTICIPATION

What actions have you taken to enable this intervention?

Prompt: How has it been made possible for midwifery staff to implement the intervention?

COLLECTIVE ACTION

How have midwifery staff responded to the roll out of the intervention?

Has it impacted on any other staff groups? If yes, explore in what way.

REFLEXIVE MONITORING

What measures have been put in place to assess the impact of the intervention and its implementation?

Is there any opportunity for feedback between stakeholders?

**TOPIC GUIDE for interview – trainers**

COHERENCE

What are you hoping to achieve through the training events?

In what way will those achievements be realised?

Prompt: In what ways does the training promote those achievements?

COGNITIVE PARTICIPATION

Do you believe in what you are doing?

Prompt: Why do you consider what you are doing important?

COLLECTIVE ACTION

How successful have the events been in helping staff to put the intervention into practice?

Prompt: Have the events been able to enthuse, engage and motivate staff to change / improve their practice and introduce this development into their work?

REFLEXIVE MONITORING

Have you been effective in your role as trainer?

Prompt: How do you know how effective you have been?
