# Supplementary material for: What helped and hindered implementation of an intervention package to reduce smoking in pregnancy: process evaluation guided by normalization process theory
Source: BMC Health Serv Res. 2019 May 9;19:297. doi: 10.1186/s12913-019-4122-1 (PMC6509824; doi:10.1186/s12913-019-4122-1)
Supplement: Supplementary file 3 — Table S5. Key themes and their relation to Normalization Process Theory concepts. (DOCX 23 kb) [file 12913_2019_4122_MOESM3_ESM.docx]

**Table S5** **Key themes and their relation to Normalization Process Theory concepts**

| **Concept** | **Application of concept** | **Findings – reported by participants** | **Cross-cutting theme** |
| --- | --- | --- | --- |
| Coherence | Evidence of how it was made to make sense | Recognised some significant changes to previous practice  Mixed levels of belief in the benefits and efficacy of the new practices  Activities were clear but allocation to roles not always clear since organisational structures varied | 1,  1, 2,  1, 2, 3 |
|  | Evidence of engagement and prioritisation by executive and senior management making it coherent | Senior management in some trusts prioritised and supported the implementation more than in others | 1, 3, |
|  | Evidence of leadership by senior managers and champions/project leads creating coherence for frontline staff | Strong partnership working in some areas whereas other areas amid re-structuring and uncertainty of future organisational structures  Staff generally took ownership of the new practices although some concerns remained | 1, 3, |
| Cognitive participation | Evidence of how staff were engaged | Internal and external sources of support legitimised the changes with some negative impacts when these were removed  Disruption to services discouraged enrolment  Perception of feasibility affected engagement  An effective champion enthused, solved problems, provided support and facilitated engagement  Easy availability of resources improved engagement | 1, 3,  1,  1, 3,  1,  4, |
|  | Evidence in relation to training | Staff were enrolled through the training  Staff were given the skills to deliver the intervention  Staff initiated and took ownership of tasks | 2,  2,  1, |
| Collective action | Evidence of what helped or hindered implementation | Workability affected actioning of tasks  Communication within and between partnering organisations impacted integration of the intervention  Adaptability to local and social context impacted integration of the intervention  Commitment demonstrated e.g. prioritisation, legitimisation  Efficient and effective data management systems  Availability of resources impacted on ability to implement | 3, 4,  1, 3,  1, 2, 3,  1, 3,  4,  4, |
| Reflexive monitoring | Evidence of appraisal of the implementation process | Trusts evidenced formal appraisal however what was measured, and how, was variable  Staff informally appraised the intervention and used it to increase/  decrease coherence, buy-in and integration | 3, 5  5, |
|  | Evidence of what the appraisal revealed | Some trusts were able to provide more appraisal evidence than others, partly due to varying levels of overall progress, as well as varying methods  Trusts struggled to establish feedback loops to staff re: i) pregnant woman’s stop smoking journey; ii) intervention’s progress | 4, 5,  3, 4, 5, |
|  | Evidence of changes made in response to reviewing progress | Trusts with higher quality data were able to review their processes more accurately and respond accordingly  Adaptation was minimised during study period to maintain protocol  Too soon to assess sustainability | 4,  5,  5. |
