# Supplementary material for: What helped and hindered implementation of an intervention package to reduce smoking in pregnancy: process evaluation guided by normalization process theory
Source: BMC Health Serv Res. 2019 May 9;19:297. doi: 10.1186/s12913-019-4122-1 (PMC6509824; doi:10.1186/s12913-019-4122-1)
Supplement: Supplementary file 4 — Table S6. Leadership and management responsible for implementation of the intervention. (DOCX 23 kb) [file 12913_2019_4122_MOESM4_ESM.docx]

**Table S6 Leadership and management responsible for implementation of the intervention**

| **Leadership** | **Number of NHS trusts** | **Intervention management** |
| --- | --- | --- |
| Stop Smoking Service led | 3 trusts | Led by key SSS manager/pregnancy specialist, but with strong links into maternity services. Generally well supported by senior maternity management. Linked into specialist SSS. Two out of three trusts had maternity care assistants who supported the SSS. |
| Maternity service led | 4 trusts | Mainly PH midwife led; sometimes there were issues about level of support from senior management; often alternative priorities (e.g. breastfeeding); often coupled with non-specialist SSS. More deviation likely from intervention service assumptions. |
| No specific lead | 1 trust | No midwife with responsibility for stop smoking or SSS pregnancy specialist linking into maternity services. |

SSS=Stop Smoking Services; PH = public health.

Three different management models were identified across the region:

- SSS-led
- Maternity-led
- No lead.

SSS-led models had a key SSPS manager/pregnancy specialist, with strong links into maternity services. This role was generally well supported by senior maternity management. Two out of three trusts using this model employed maternity care assistants who supported the SSPS. These Trusts were perceived to adhere to the protocol with higher levels of fidelity compared to other models. Maternity-led models existed in 4 Trusts. They were usually led by a public health midwife; sometimes there were issues about level of support from senior maternity management who were reported to have alternative priorities (e.g. breastfeeding). This model was often coupled with non-specialist SSS. More deviation from intervention service assumptions was reported compared to SSS-led models. One Trust was reported to have no lead. There was no midwife with responsibility for stop smoking and no stop smoking specialist linking into maternity services. This Trust was reported to struggle to implement the pathway and to keep its progress under review i.e. there was minimal ‘reflexive monitoring’. Monitoring requires patent communication channels and SSS-led models were reported to have created these most successfully.
