# Supplementary material for: What helped and hindered implementation of an intervention package to reduce smoking in pregnancy: process evaluation guided by normalization process theory
Source: BMC Health Serv Res. 2019 May 9;19:297. doi: 10.1186/s12913-019-4122-1 (PMC6509824; doi:10.1186/s12913-019-4122-1)
Supplement: Supplementary file 5 — Table S7. Key resources and organisational variance. (DOCX 22 kb) [file 12913_2019_4122_MOESM5_ESM.docx]

**Table S7 Key resources and organisational variance**

| **Resource** | **Necessary for full implementation** | **‘Best practice’ collective actions** | **Comment** |
| --- | --- | --- | --- |
| Training | Mandatory for core delivery staff (i.e. maternity and SSPS staff). | Made available to core intervention staff **and** a wide variety of related staff.  Made accessible from multiple work sites, offered in a continuous cycle of updates and to new employees. | Training less accessible where staff had to travel long distances to the venue.  More frequent updates required than anticipated to maintain training standard. |
| CO monitors | Monitors that were fit for purpose (e.g. high visual impact, no calibration needed, suitable for clinical use, available in sufficient numbers).  Monitors bought, distributed, maintained and repaired in a reliable and timely manner. | Developed system that a) took over supply of mouthpieces and non-alcoholic wipes for ongoing use of the monitors through efficient re-ordering of consumables and b) ensured timely maintenance and repair. | Efficiency of re-ordering and maintenance systems varied.  Generic issues with new CO monitors created challenges (e.g. batteries ran down more quickly than previous versions, sensitive to cold places like car boots and contamination with alcohol gel). |
| Referral forms | Forms that reflected the requirements of the new pathway and that made smoking status clear to enable performance management. | Forms were further adapted to the organisations to give more, local data.  Specific systems set up to provide feedback loops. | Reports of low standard of form completion were widespread.  Feedback loops to let maternity staff know which women were picking up on the stop smoking offer were only established successfully in some trusts. |
| RPT computer software and fetal doll | Provision of standardised equipment.  Training to use the RPT equipment.  Sufficient numbers of midwives trained to use the RPT equipment. | Planning of the logistics to offer the RPT (e.g. time, place, concurrent with dating scan clinic, link into SC follow up, skill-mix, staff availability). | Variation in numbers of staff identified for training and considered to be adequately trained.  RPT implemented within different timeframes. It was logistically challenging (e.g. locating a computer in a room close to the scanner, running concurrently with a scan clinic attended by pregnant smokers, to ensure that the RPT was available to more smokers/recent quitters, providing stop smoking follow-up as soon as possible). RPT-trained midwives were only available at limited times. |
| Publicity | Provision of SSPS leaflets and posters, contextualised to local systems, at an acceptable cost. | Additional, organisation-specific leaflets. | The cost of printing additional leaflets was considered prohibitive, especially as changes meant frequent updating was required. |
| Intervention setting | Intervention setting and SSPS follow up options. | Availability of options where women made early contact with the maternity service and received SSPS follow up (e.g. early bird (pre-booking) clinic, first booking appointment, antenatal clinic, community clinic, home). | SSPS delivery models varied widely, some offering more intervention setting and follow up options than others. |
| Staff availability | Identification of staff to deliver intervention. | Adequate numbers of staff (maternity, SSS and other SSPS providers) with appropriate expertise: required at each point in the pathway; depending on service model this was easier to comply with in some organisations. | The actual staff numbers and allocation of roles/responsibilities varied and was dependent on local maternity and stop smoking service delivery models and allocation of funding. |
| Staff time | Release of staff for training and to deliver intervention. | To allow back fill for training, release to carry out RPT, extra time spent giving advice, allowance for high rates of women ‘unable to contact’ and non-attendance at SC appointments, extra time spent following up on the telephone offering appointments, extra follow up visits and ‘hand-holding’ throughout the pathway. | There were hidden impacts on staff time, especially to implement the RPT and during SSPS follow up.  Level of implementation support varied and was dependent on local maternity and SSPS delivery models and allocation of funding. |
| Data management system | System for data management across organisations. | An efficient system (e.g. QuitManager^©^ ensured continuing support from funders and managers and allowed easy, electronic access to data and simple generation of monitoring reports). | Wide variability in type, functionality and capacity. Essential to allow for audit of the progress of the intervention package.  Not available in some areas. |

CO = carbon monoxide; RPT = risk perception tool; SSPS = stop smoking in pregnancy services; SSS=Stop Smoking Services; SC=smoking cessation.
